# The association between serum orexin A and short‐term neurological improvement in patients with mild to moderate acute ischemic stroke

**DOI:** 10.1002/brb3.2845

**Published:** 2022-12-27

**Authors:** Shiyu Hu, Lijie Ren, Yang Wang, Zhihao Lei, Jingjing Cai, Suyue Pan

**Affiliations:** ^1^ Nanfang Hospital, Southern Medical University/The First School of Clinical Medicine, Southern Medical University Guangzhou China; ^2^ Neurology Department of Shenzhen Second People's Hospital/First Affiliated Hospital of Shenzhen University Health Science Center Shenzhen China

**Keywords:** acute ischemic stroke, biomarker, orexin A, short‐term neurological improvement

## Abstract

**Background:**

The serum orexin A level was significantly lower among patients with acute ischemic stroke (AIS) and negatively related to the volume of the infarction, but the relationship between serum orexin A and prognosis of AIS was still unclear. We aimed to clarify the association between serum orexin A and the short‐term neurological improvement in patients with mild to moderate AIS.

**Methods:**

We consecutively enrolled patients with first ever mild to moderate AIS admitted to hospital within 48 h from symptom onset in this prospective observational study. The serum orexin A concentrations were determined on the second morning since the admission. The short‐term neurological improvement was defined as more than 1 point decrease in the National Institute of Health Stroke Scale score within 7 days after admission.

**Results:**

We detected increased serum orexin A level in mild to moderate AIS patients with early onset of stroke‐related insomnia (33.44 vs 18.66 pg/ml, *p* = .004) as well as in patients with short‐term neurological improvement compared to those without improvement (31.78 vs 16.24 pg/ml, *p* = .038). The serum orexin A level was positively associated with the short‐term neurological improvement after adjusting for sleep condition and other related variables.

**Conclusion:**

Serum orexin A might be a useful biomarker for the assessment of early prognosis in patients with mild to moderate AIS.

## Graphical abstract

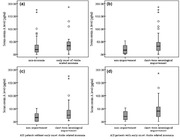

## 1 INTRODUCTION

Orexin A, also called hypocretin‐1, is a neuropeptide produced by the lateral hypothalamus, and it exerts its biological functions through activation of orexin‐1 (OX1R) and orexin‐2 (OX2R) receptors (Sakurai et al., 1998). Increased serum levels of orexin A could induce insomnia since they stabilize arousal (Mahler et al., 2014; Sakurai, 2007). Selective dual orexin receptor antagonists (DORAs) are now accepted as one of the pharmacological therapies for patients with insomnia (Coleman et al., 2017). Thus, increased serum orexin A levels in acute ischemic stroke (AIS) patients might induce a higher risk of poststroke insomnia, which is detrimental to neurological recovery based on previous studies (Ho et al., 2021; Suh et al., 2016). In contrast, orexin A showed neuroprotective effects on rats with cerebral ischemia and hypoxia (Kitamura et al., 2010; Sokołowska et al., 2012).

In a human study, the serum orexin A level was significantly lower among patients with AIS, and the cerebrospinal fluid was inversely associated with brain infarct volumes (Kotan et al., 2013). However, theoretically, decreased serum orexin A could induce a lower risk of stroke‐related insomnia, which might be beneficial to neurological recovery, especially for patients with mild to moderate AIS. Thus, the effect of orexin A on sleep condition and neurological protection was kind of contradictory. To date, the relationship between serum orexin A levels and short‐term neurological function in patients with AIS remains unclear. The aim of this study was to clarify the association between serum orexin A and short‐term neurological improvement in patients with mild to moderate AIS.

## 2 METHODS

### 2.1 Study population

We prospectively recruited consecutive patients with first ever mild to moderate AIS admitted to the Neurology Department of Shenzhen Second People's Hospital within 48 h of symptom onset from January 2020 to June 2021. We enrolled patients with mild to moderate stroke who had a modified Rankin Scale (mRS) score ≤3. We excluded patients with the following conditions: (1) age < 18; (2) isolated visual changes, sensory symptoms (such as numbness), dizziness or vertigo, and no evidence of AIS on brain CT or MRI; (3) intravenous thrombolysis or endovascular therapy; (4) scheduled for surgery within the following 3 months; (5) anxiety or depression or chronic insomnia; (6) malignant tumor or other diseases with life expectancy < 3 months; (7) respiratory and circulatory failure (heart function grade of NYHA was III or IV with/without oxyhemoglobin saturation less than 60%); and (8) unable to be followed up due to severe cognitive impairment or any other condition. We performed this observational study in accordance with the relevant guidelines and regulations. Clinical management, including antiplatelet therapy, statins, and antihypertensive therapy, was performed with the AHA/ASA guidelines for all enrolled patients with mild to moderate AIS (Powers et al., 2019).

### 2.2 Clinical assessment

Data recorded included age, sex, body mass index, smoking status, alcohol intake, National Institute of Health Stroke Scale (NIHSS) score, and mRS score at admission and discharge, history of hypertension (systolic blood pressure, SBP > 140 mmHg or diastolic blood pressure, DBP > 90 mmHg at admission, self‐reported history of hypertension or regularly taking oral antihypertensive agents), diabetes (including self‐reported history of diabetes or regularly taking oral hypoglycemic agents or insulin injections), coronary heart disease, and obstructive sleep apnea‐hypopnea syndrome (diagnosed by polysomnography or other qualified sleep monitoring instrument). Mean blood pressure was defined as the average monitored SBP and DBP value during hospitalization. The subtype of AIS was assessed according to the Trial of Org 10 172 in Acute Stroke Treatment criteria (Adams et al., 1993).

Except for the serum orexin A concentration, other blood laboratory tests, including hypersensitive C reactive protein, urine acid, creatinine, alanine transaminase, fasting blood glucose, glycosylated hemoglobin, total cholesterol, triglyceride, low‐density lipid cholesterol, high‐density lipid cholesterol, and homocysteine, were collected and assessed after an 8‐h overnight fasting venous blood sample. The postprandial blood glucose level was assessed 2 h after breakfast.

### 2.3 Serum orexin A level detection

Blood samples were collected with EDTA tubes at 7 a.m. on the second morning of admission after 8 h of overnight fasting and keeping in bed. Then, the whole blood sample was coagulated at room temperature for 20 min followed by centrifugation at 2000–3000 r.p.m. for 20 min to produce serum. The serum orexin A level was measured with the enzyme linked immunoassay double antibody method and compared to the O.D. at a wavelength of 450 nm of the samples to a standard curve (assay range from 12.5 to 400 pg/ml with minimum detectable dose of 1.0 pg/ml). Reagents were provided by Huinuo Biological Technology, Shenzhen, China. The assessments were blinded with respect to the clinical data.

### 2.4 Clinical evaluation

At baseline, we used the Athens Insomnia Scale (AIS) to evaluate the incidence of stroke‐related insomnia. Early onset stroke‐related insomnia was defined as an AIS score > 5 points (Szelenberger & Niemcewicz, 2000). Sleep quality was measured by the Pittsburgh Sleep Quality Index. The Hamilton Rating Scale for Depression (HAM‐D) and Anxiety (HAM‐A) were used to evaluate whether there were combined depressive (HAM‐D > 17) or anxiety (HAM‐D > 14) disorders. Based on previous studies, we defined short‐term neurological improvement as a more than 1‐point decrease (ΔNIHSS ≥ 2 points) or a decrease to zero in the NIHSS score within 7 days after admission for patients with mild to moderate AIS (Irie et al., 2015; Muscari et al., 2013).

### 2.5 Statistical analysis

We presented continuous variables as the mean ± standard deviation or medians (interquartile range) based on data distribution. Categorical variables are presented as frequencies. Differences between groups were compared by means of the *t‐*test for normally distributed continuous variables and the Kruskal–Wallis nonparametric test for nonnormally distributed variables. Pearson's chi‐squared test was used for the comparison of categorical variables. Multivariate binary logistic regression analysis was used to assess the predictors of short‐term neurological improvement, while variables with *p‐*value < .15 in univariable binary logistic regression analysis were used for adjustment. All data were fully available without restriction. Statistical analysis was performed using SPSS software, version 22.0 (SPSS IBM Inc, Chicago, IL, USA) and STATA version 15 (StataCorp LLC, College Station, TX, USA), and statistical significance was considered when a two‐tailed *p* < .05.

## 3 RESULTS

**FIGURE 1:**
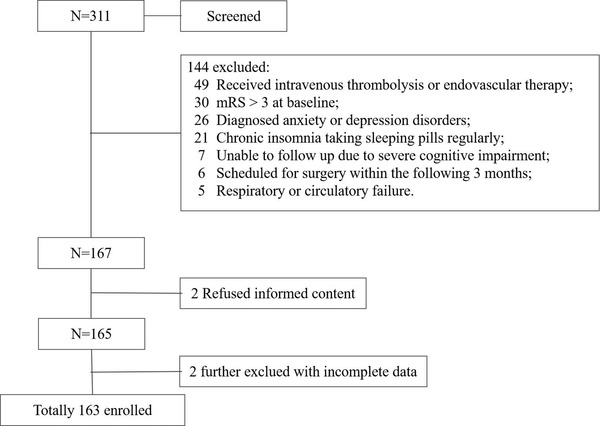
Patients selected flow chart. The flow chart shows the selection process by which the patients were submitted to statistical analysis

Overall, we enrolled 163 patients with first‐ever mild to moderate AIS (Figure 1). Their mean age was 62, and 66.3% were male. Among them, 87 (53.4%) showed short‐term neurological improvement on day 7. Patients with short‐term neurological improvement were younger (60 vs. 64, *p* = .143) than those without improvement. The median NIHSS score on admission (3 vs. 2, *p* = .014) was slightly higher in the group with neurological improvement. Detailed baseline characteristics of the study population are presented in Table 1. There were more patients suffering early onset stroke‐related insomnia (41.4 vs. 25%, *p* = .027) in patients with short‐term neurological improvement, but sleep quality and sleep efficiency showed no significant difference between patients with or without insomnia. The overall neurological outcome was good in the study population, and the NIHSS score was lower in patients with short‐term neurological improvement (1 vs. 2, *p* = .004) (details shown in Table 2). Overall, most of the stroke subtype of the enrolled patients was small arterial occlusion (SAO), followed by large arterial atherosclerosis (LAA), stroke of undetermined etiology, cardioembolism, and stroke of other determined etiology. There was no significant difference in the stroke subtype distribution between patients with or without short‐term neurological improvement (*p* = .743) (see Supporting Information 1 for details).

**TABLE 1:** Baseline characteristics of enrolled mild to moderate AIS patients with or without short‐term neurological improvement

	Neurological improvement (*n* = 87)	Non‐improvement (*n* = 76)	*p* value*
Age (years)	60 [51–66]	64 [54–69]	.143
Male (%)	67.8	64.5	.653
BMI (kg/m^2^)	24.74 ± 3.53	24.80 ± 2.85	.906
Currently smoking (%)	50.6	42.1	.280
History of diseases			
Hypertension (%)	57.5	63.2	.459
Diabetes mellitus (%)	28.7	34.2	.452
CHD (%)	6.9	10.5	.409
OSAHS (%)	4.6	1.3	.373
Admission NIHSS	3 (Sakurai, 2007; Mahler et al., 2014; Coleman et al., 2017)	2 (Sakurai et al., 1998; Sakurai, 2007; Mahler et al., 2014)	.014
Admission mRS	2 (Sakurai et al., 1998; Sakurai, 2007; Mahler et al., 2014)	2 (Sakurai et al., 1998; Sakurai, 2007)	.326
Mean SBP (mmHg)	138 ± 14	139 ± 13	.825
Mean DBP (mmHg)	81 [76–87]	81 [76–86]	.956
Laboratory assessments			
hsCRP (mmol/L)	1.23 [0.50–2.23]	1.14 [0.50–2.50]	.543
Hb (g/L)	140.0 [131.0–153.0]	136.0 [127.5–145.8]	.103
ALT (U/L)	18.80 [13.00–26.10]	18.95 [13.30–23.85]	.808
Cr (umol/l)	70.20 [60.10–80.00]	68.90 [57.18–79.30]	.758
UA (umol/l)	358.61 ± 94.83	338.35 ± 98.97	.188
TG (mmol/l)	1.33 [0.97–2.15]	1.44 [1.14–2.01]	.799
TC (mmol/l)	4.55 [3.81–5.42]	4.47 [3.76–5.33]	.268
LDL‐C (mmol/l)	2.78 [2.24–3.45]	2.58 [2.06–3.45]	.143
HDL‐C (mmol/l)	1.03 ± 0.24	1.06 ± 0.24	.445
HCY (umol/l)	11.20 [9.20–13.30]	10.45 [8.70–13.80]	.513
FBG (mmol/l)	5.20 [4.68–7.04]	5.33 [4.80–6.90]	.304
PBG (mmol/l)	7.47 [6.54–10.68]	7.85 [6.57–12.18]	.096

Abbreviations: BMI, body mass index; CHD, coronary heart disease; HDL‐C, high density lipoprotein cholesterol; LDL‐C, low‐density lipoprotein cholesterol; HCY, homocysteine; FBG, fasting blood glucose; HbA1C, glycosylated hemoglobin; PBG, postprandial blood glucose; OSAHS, obstructive sleep apnea‐hypopnea syndrome; NIHSS, National Institutes of Health Stroke Scale; mRS, modified Rankin Scale; SBP, systolic blood pressure; DBP, diastolic blood pressure; Hcrt, hypocretin; hsCRP, hypersensitive C reacted protein; ALT, alanine aminotransferase; Cr, creatine; UA, uric acid; TC, total cholesterol; TG, triglyceride.

* *p* < .05 was statistically significant.

**TABLE 2:** Sleep and neurological evaluation on day 7 since disease onset among mild to moderate AIS patients with or without short‐term neurological improvement

	Neurological improvement (*n* = 87)	Non‐improvement (*n* = 76)	*p* value*
Sleep condition			
Stroke related insomnia (%)	41.4	25.0	.027
SE (%)	88.80 [83.30–93.75]	89.45 [85.70–100.00]	.352
PSQI	7 (Mahler et al., 2014; Coleman et al., 2017; Suh et al., 2016; Ho et al., 2021; Kitamura et al., 2010; Sokołowska et al., 2012; Kotan et al., 2013; Powers et al., 2019; Adams et al., 1993; Szelenberger & Niemcewicz, 2000)	7 (Mahler et al., 2014; Coleman et al., 2017; Suh et al., 2016; Ho et al., 2021; Kitamura et al., 2010; Sokołowska et al., 2012; Kotan et al., 2013; Powers et al., 2019)	.547
HAM‐A score	3 [0–6]	2 (Sakurai et al., 1998; Sakurai, 2007; Mahler et al., 2014; Coleman et al., 2017; Suh et al., 2016)	.132
HAM‐D score	3 [0–6]	1 [0–4]	0.104
Neurological function			
NIHSS score	1 (Sakurai et al., 1998; Sakurai, 2007)	2 (Sakurai et al., 1998; Sakurai, 2007; Mahler et al., 2014)	.004
mRS score	1 (Sakurai et al., 1998; Sakurai, 2007)	1.5 (Sakurai et al., 1998; Sakurai, 2007)	.005

Abbreviation: SE, sleep efficiency; PSQI, Pittsburgh Sleep Quality Index; HAM‐A, Hamilton Rating Scale for Anxiety; HAM‐D, Hamilton Rating Scale for Depression; NIHSS, National Institutes of Health Stroke Scale; mRS, modified Rankin Scale.

* *p* < .05 was statistically significant.

**FIGURE 2:**
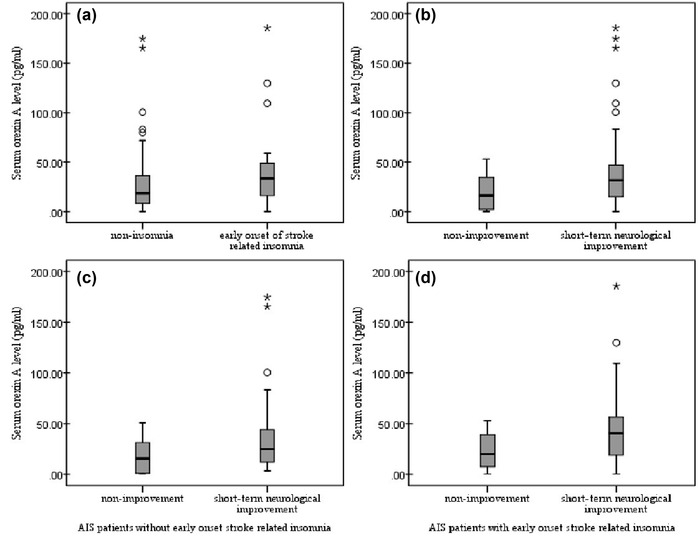
The serum orexin A level in patients with mild to moderate AIS. The serum orexin A level was significantly increased in AIS patients with early onset of stroke related insomnia (a) (31.78 vs 16.24 pg/ml, *p* = .038), as well as in AIS patients with short‐term neurological improvement (b) (33.44 vs 18.66 pg/ml, *p* = .004). In patients without early onset stroke related insomnia (c), the difference of serum orexin A level was slightly reduced between patients with or without short‐term neurological improvement (24.65 vs 15.36 pg/ml, *p* = .376), while a higher serum orexin A level was detected in patients with early onset stroke related insomnia (d) if they got short‐term neurological improvement (40.38 vs 19.84 pg/ml, *p* = .162), but the difference was not statistical significant in the this further analysis (the outliers greater than 200 pg/ml have been removed in the boxplots)

For the serum orexin A level assessment, the mean serum orexin A level was 23.99 pg/ml among patients with mild to moderate AIS. We detected increased serum orexin A levels (31.78 vs. 16.24 pg/ml, *p* = .038) in patients with short‐term neurological improvement. Additionally, the serum level of orexin A was also higher (33.44 vs. 18.66 pg/ml, *p* = .004) in patients with early‐onset stroke‐related insomnia. In patients with early‐onset stroke‐related insomnia, the serum orexin A level was even higher if they experienced short‐term neurological improvement (40.38 vs. 19.84 pg/ml), although the difference was not statistically significant (*p* = .162) (details shown in Figure 2).

**FIGURE 3:**
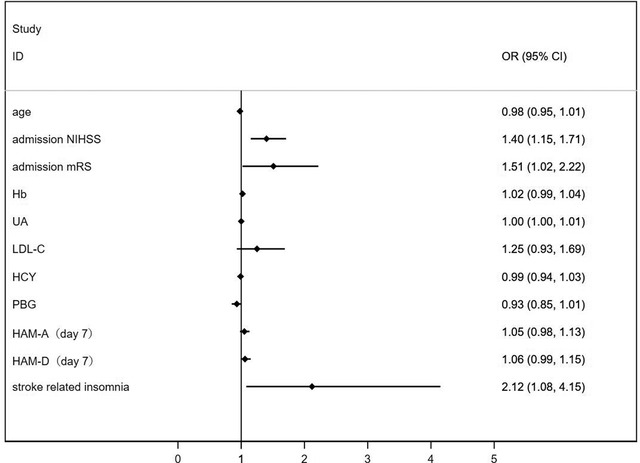
The odds ratio (OR) and 95% confidence interval (CI) of adjusted confounding factors ultimately enrolled in the multivariate binary logistic regression analysis

In the multivariate binary logistic regression analysis, the serum orexin A level was positively associated with short‐term neurological improvement (per SD increment, adjusted OR: 1.65, 95% CI: 1.03–2.63, *p* = 0.036) after adjusting for the incidence of stroke‐related insomnia, admission NIHSS score, admission mRS score, HAM‐A score, and HAM‐D score on day 7 as well as other traditional related factors related to neurological recovery (Figure 3 and Table 3; details of the univariate binary logistic regression analysis are shown in Supporting Information 2). Consistently, when serum orexin A was assessed as quartiles, a significantly higher incidence of short‐term neurological improvement was found among participants in quartile 4 (≥ 41.40 pg/ml) compared with participants in quartile 1 (< 10.63 pg/ml) after adjusting for possible confounders. Specifically, the adjusted OR of quartile 4 was 7.4 (95% CI: 2.35–23.33, *p* = .001) (*p*‐trend = .008) (Table 3).

**TABLE 3:** Association of serum orexin A with short‐term neurological improvement

		Crude model^a^		Adjusted model ^b^	
Orexin A, (pg/ml)	Cases (%)	OR [95% CI]	*p* value*	OR [95% CI]	*p* value*
Short‐term neurological improvement					
Per SD increment		1.53 [0.98–2.40]	.064	1.65 [1.03–2.63]	.036
Quartile 1	12 (30.0)	ref		ref	
Quartile 2	23 (56.1)	2.98 [1.94–7.45]	.019	2.18 [0.80–5.91]	.127
Quartile 3	21 (51.2)	2.45 [0.98–6.10]	.054	1.98 [0.72–5.48]	.186
Quartile 4	31 (75.6)	7.23 [2.71–19.32]	< .001	7.40 [2.35–23.33]	.001
*p* for trend			.001		.008

Abbreviation: BMI, body mass index; SBP, systolic blood pressure; DBP, diastolic blood pressure; NIHSS, National Institutes of Health Stroke Scale; HAM‐A, Hamilton Rating Scale for Anxiety; AIS, Athens Insomnia Scale; FBG, fasting blood glucose; LDL‐C, low‐density lipoprotein cholesterol; UA, uric acid; HCY, homocysteine.

^a^ Univariate.

^b^ Adjusted for age, BMI, hypertension history, mean SBP, mean DBP, HAMA score, admission NIHSS score, AIS score, FBG, LDL‐C, UA and HCY level.

* *p* < .05 was statistically significant.

**FIGURE 4:**
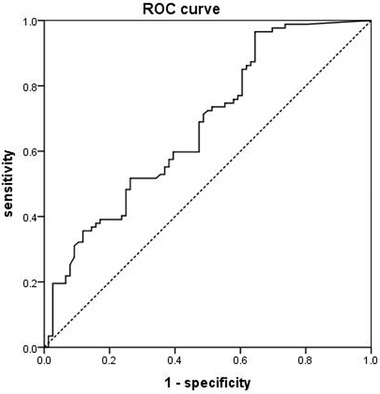
ROC curve in AIS patients showing the performance of serum orexin A in predicting the short‐term neurological improvement (Area under curve = 0.678, *p* < .001)

The area under the ROC curves predicted a short‐term neurological improvement in AIS patients based on serum orexin A levels. The AUC for short‐term neurological improvement was 0.678 (*p* < .001), which corresponds to a serum orexin A cutoff value of 16.87 pg/ml (96.6% sensitivity and 35.5% specificity) based on the Youden Index (Figure 4). When serum orexin A was assessed as quartiles, the baseline information showed that the serum HCY level was higher among patients in quartile 4 (12.20 μmol/l/l). After pairwise comparisons, we found significantly increased serum HCY levels in quartile 4 compared with quartiles 1 to 3 (Supporting Information 3).

## 4 DISCUSSION

Stroke is the second leading cause of disability worldwide and the leading cause of death in China, with a recurrence rate up to 6.7% (Ma et al., 2021; Roth et al., 2020; Tu et al., 2021). Therefore, exploring any factor that might influence disease prognosis is essential to the management of stroke.

In our study, compared with populations without AIS in the research conducted by Tang S et al., the mean serum orexin A level was lower in our study subjects, consistent with previous findings (Kotan et al., 2013; Tang et al., 2017). A relatively increased serum orexin A was detected in AIS patients with short‐term neurological improvement compared with no improvement patients in this study. In the logistic analysis, we found that serum orexin A was positively related to short‐term neurological improvement in AIS patients. The positive relationship was still significant after adjusting for sleep condition and other possible confounders.

As we mentioned earlier, decreased serum orexin A levels were found to be associated with the aggravation of atherosclerosis, which offered a partial explanation of the protective effect of orexin A on the progression of AIS (Mcalpine et al., 2019). However, a majority of the stroke subtype was SAO instead of LAA among the enrolled patients. Therefore, orexin A also plays a key role in promoting neurological improvement through pathways other than the inhibition of atherosclerosis in AIS patients (Sikder & Kodadek, 2007; Xu et al., 2021). Additionally, a study of MCAO rats suggested that orexin A could reduce neuronal apoptosis by inhibiting overactivated autophagy through the OX1R‐mediated MAPK/ERK/mTOR pathway, consequently playing a neuroprotective role under cerebral ischemic conditions (Xu et al., 2021).

It seems surprising that more patients complained of stroke‐related insomnia in the short‐term neurological improvement group, but there was no difference in sleep quality and sleep efficiency between the two groups. Increased serum orexin levels might be the most likely interference. We also detected a significantly increased plasma homocysteine level corresponding to the highest serum orexin A concentrations compared with the lowest group. Contrary to the protective effects of serum orexin A in AIS, a serum level of homocysteine > 10.3 μmol/L was found to be associated with early neurological deterioration in AIS patients (Kwon et al., 2014). We proposed that the protective effects were in the early progression of AIS. The causal effects between serum orexin A and homocysteine need to be confirmed by further experiments.

Although there was no significant difference in age among AIS patients in different serum orexin A level quartiles in the present study, earlier studies showed decreased levels of orexin during the aging process. However, serum orexin levels have been shown to increase with aging (Hunt et al., 2015; Matsumura et al., 2002). Since aging is a widely accepted traditional risk factor for AIS, increased serum orexin A levels might provide some protection against stroke in the elderly population. We suggest that the clinical application of DORA for the management of insomnia in patients with mild to moderate AIS should be done with caution based on our findings.

There were several limitations in this study. First, we only detected the serum concentrations of orexin A instead of the overall orexins. This was because orexin A activates both OX1R and OX2R receptors and exerts its biological functions as hypocretins on the progression of atherosclerosis diseases. The role of orexin B in the atherosclerotic process needs to be further explored. Second, we only assessed baseline serum orexin A concentrations for the AIS patients. The relationship between serum orexin A and short‐term neurological improvement might be better illustrated if serum orexin A levels were tested at different time points during hospitalization. Furthermore, retesting of serum orexin A during follow‐up might be useful to assess the relationship between serum orexin A and the long‐term neurological function of patients with AIS in future studies. Third, the median NIHSS score was relatively low in this study, since we only enrolled AIS patients with mRS ≤ 3 to minimize the effect of time in bed on sleep condition. Orexin A also plays an important role in sleep regulation. For real‐world research, we will involve patients without such strict restriction on the admission NIHSS score in our future work. Fourth, the overall sample size was small. Our findings should be interpreted with caution since many covariates have been included in the regression models. Residual confounding effects from unmeasured factors cannot be excluded. The specificity of orexin A in the ROC curve was relatively low. We performed our study according to the STROBE guidelines, but a discovery or validation cohort was lacking because of the small study sample size. Therefore, additional large sample studies are needed to further examine the association of serum orexin A and short‐term neurological improvement in AIS patients.

## 5 CONCLUSION

We observed that increased serum orexin A levels in non‐bedridden AIS patients were associated with short‐term neurological improvement. Orexin A might be a valuable predictor of early prognosis in patients with mild to moderate AIS.

## AUTHOR CONTRIBUTIONS

Yang Wang, Lijie Ren, and Shiyu Hu conceived and designed the study. Zhihao Lei and Shiyu Hu collected the data. Shiyu Hu performed the statistical analysis and wrote the paper. Jingjing Cai assessed the serum orexin A concentrations. Suyue Pan reviewed and edited the manuscript. All authors read and approved the manuscript.

## CONFLICT OF INTEREST

The authors declare no conflict of interest to disclose.

### PEER REVIEW

The peer review history for this article is available at https://publons.com/publon/10.1002/brb3.2845

## Supporting information

Supporting Information

## Data Availability

The data analyzed during the current study available from the corresponding author on reasonable request.
