# Supplementary material for: The association between serum orexin A and short‐term neurological improvement in patients with mild to moderate acute ischemic stroke
Source: Brain Behav. 2022 Dec 27;13(1):e2845. doi: 10.1002/brb3.2845 (PMC9847589; doi:10.1002/brb3.2845)
Supplement: Supplementary file 1 — Supporting Information [file BRB3-13-e2845-s001.docx]

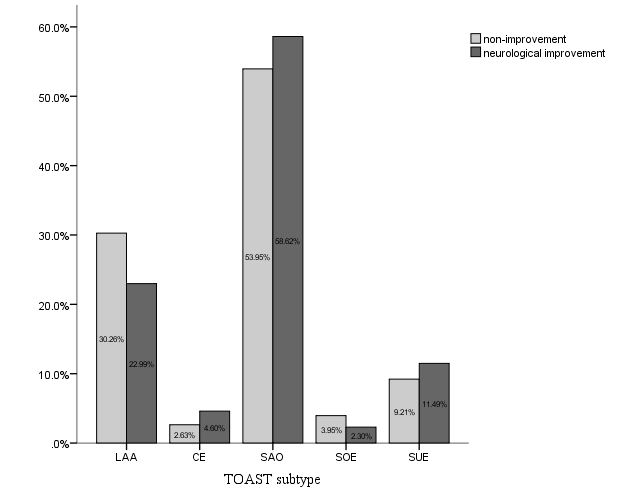


Supplement 1. The TOAST subtype of mild to moderate AIS patients with or without short-term neurological improvement.

There was no significant difference in the stroke subtype distribution between patient with or without short-term neurological improvement (p=0.743)

|  | Univariate model | p value* |
| --- | --- | --- |
|  | OR [95% CI] |  |
| Short-term neurological improvement | | |
| Age (yrs) | 0.98 [0.95-1.01] | 0.144 |
| Male (ref=female ) | 0.86 [0.45-1.65] | 0.653 |
| BMI | 0.99 [0.90-1.09] | 0.905 |
| Currently smoking  (ref=ever or never smoke) | 1.41 [0.76-2.62] | 0.280 |
| History of diseases | 0.77 [0.32-1.87] | 0.566 |
| Hypertension | 0.79 [0.42-1.48] | 0.460 |
| Diabetes mellitus | 0.78 [0.40-1.51] | 0.452 |
| CHD | 0.63 [0.21-1.90] | 0.413 |
| OSAHS (%) | 3.61 [0.40-33.06] | 0.255 |
| Admission NIHSS | 1.40 [1.15-1.71] | 0.001 |
| Admission mRS | 1.51 [1.02-2.22] | 0.037 |
| Mean SBP | 0.99 [0.98-1.02] | 0.997 |
| Mean DBP | 1.00 [0.96-1.04] | 0.956 |
| Sleep and emotional conditions |  |  |
| HAM-A score | 1.05 [0.98-1.13] | 0.135 |
| HAM-D score | 1.06 [0.99-1.15] | 0.107 |
| Stroke related insomnia | 2.12 [1.08-4.15] | 0.029 |
| PSQI score | 1.01 [0.97-1.06] | 0.545 |
| Sleep efficiency (%) | 0.99 [0.96-1.02] | 0.352 |
| Laboratory assessments |  |  |
| hsCRP (mmol/l) | 0.99 [0.95-1.03] | 0.547 |
| Hb (g/L) | 1.02 [0.99-1.04] | 0.104 |
| ALT (U/L) | 1.00 [0.98-1.02] | 0.807 |
| Cr (umol/l) | 1.00 [0.99-1.02] | 0.757 |
| UA (umol/l) | 1.00 [1.00-1.01] | 0.189 |
| TG (mmol/l) | 0.97 [0.76-1.24] | 0.798 |
| TC (mmol/l) | 1.15 [0.90-1.46] | 0.268 |
| LDL-C (mmol/l) | 1.25 [0.93-1.69] | 0.145 |
| HDL-C (mmol/l) | 0.61 [0.17-2.19] | 0.443 |
| HCY (umol/l) | 0.99 [0.94-1.03] | 0.518 |
| FBG (mmol/l) | 0.93 [0.80-1.07] | 0.305 |
| HbA1C (%) | 0.97 [0.80-1.17] | 0.724 |
| PBG (mmol/l) | 0.93 [0.85-1.01] | 0.098 |

Supplement 2.Univariate binary logistic regression analysis of factors associated with short-term neurological improvement

Abbreviations: BMI: body mass index, CHD: [coronary heart disease](C:/Program%20Files%20(x86)/Youdao/Dict/8.9.6.0/resultui/html/index.html#/javascript:;), OSAHS: obstructive sleep apnea-hypopnea syndrome, NIHSS: National Institutes of Health Stroke Scale, mRS: modified Rankin Scale, AIS: Athens Insomnia Scale, PSQI: Pittsburgh Sleep Quality Index, HAM-A: Hamilton Rating Scale for Anxiety, HAM-D: Hamilton Rating Scale for Depression, SBP: systolic blood pressure, DBP: diastolic blood pressure, hsCRP: hypersensitive C reacted protein, ALT: alanine aminotransferase, Cr: creatine, UA: uric acid, TC: total cholesterol, TG: triglyceride; HDL-C: high density lipoprotein cholesterol; LDL-C: low-density lipoprotein cholesterol, HCY: homocysteine, FBG: fasting blood glucose, HbA1C: glycosylated hemoglobin, PBG: postprandial blood glucose.

*p<0.05 was statistical significant.

Supplement 3. Baseline sleep and atherosclerosis related factors in AIS patients with different quartile serum orexin A level

|  | Serum orexin A level (pg/ml) | | | | p value |
| --- | --- | --- | --- | --- | --- |
|  | Q1 (<10.63) | Q2 (10.63 -23.99) | Q3 (23.99 to <40.90) | Q4 (≥40.90) |  |
| Age (year) | 64 [54-70] | 62 [56-68] | 55 [50-67] | 60 [53-71] | 0.221 |
| Female (%) | 40.0 | 34.1 | 31.7 | 29.3 | 0.766 |
| BMI，kg/m2 | 25.16 [23.19-26.64] | 24.22 [22.39-26.01] | 24.36 [22.89-26.39] | 24.63 [21.56-27.84] | 0.863 |
| Current smoking (%) | 35.0 | 41.5 | 53.7 | 56.1 | 0.179 |
| Alcohol (%) | 10.0 | 17.1 | 9.8 | 19.5 | 0.478 |
| HTN (%) | 67.5 | 56.1 | 51.2 | 65.9 | 0.376 |
| DM (%) | 35.0 | 36.6 | 29.3 | 24.4 | 0.620 |
| CHD (%) | 7.5 | 12.2 | 2.4 | 12.2 | 0.335 |
| OSAHS (%) | 2.5 | 2.4 | 2.4 | 4.9 | 0.895 |
| Admission NIHSS score | 2 [1-3] | 3 [2-5] | 2 [1-4] | 2 [2-4] | 0.089 |
| Admission mRS score | 2 [1-2] | 2 [1-3] | 2 [1-2] | 2 [1-2] | 0.560 |
| Mean SBP (mmHg) | 140 [136-147] | 137 [125-150] | 140 [127-147] | 138 [128-152] | 0.512 |
| Mean DBP (mmHg) | 83 [77-86] | 80 [76-85] | 80 [76.-86] | 83 [77-89] | 0.278 |
| Laboratory assessments |  |  |  |  |  |
| hsCRP (mmol/l) | 1.15 [0.56-2.24] | 1.02 [0.50-2.65] | 1.31 [0.50-3.61] | 1.05 [0.50-1.99] | 0.681 |
| Hb (g/L) | 135.0 [126.0-149.0] | 138.0 [128.0-154.5] | 139.0 [132.3-148.8] | 138.5 [132.0-154.8] | 0.304 |
| ALT (U/L) | 19.30 [14.70-28.00] | 18.00 [12.55-25.00] | 19.95 [14.00-25.25] | 16.00 [12.43-23.88] | 0.408 |
| Cr (umol/l) | 65.00 [55.10-80.70] | 70.70 [59.20-85.10] | 72.95 [58.90-77.95] | 70.00 [61.83-78.65] | 0.828 |
| UA (umol/l) | 338.40 [249.40-390.70] | 329.40 [269.25-399.30] | 336.75 [276.63-417.03] | 364.35 [299.10-419.20] | 0.471 |
| TG (mmol/l) | 1.60 [1.12-2.19] | 1.33 [1.09-1.80] | 1.46 [1.08-2.11] | 1.33 [0.95-2.17] | 0.253 |
| TC (mmol/l) | 4.55 [3.84-5.90] | 4.68 [3.69-5.31] | 4.68 [4.06-5.40] | 4.41 [3.71-5.30] | 0.909 |
| LDL-C (mmol/l) | 2.64 [2.24-3.59] | 2.94 [2.31-3.55] | 2.76 [2.32-3.41] | 2.68 [2.05-3.26] | 0.852 |
| HDL-C (mmol/l) | 0.97 [0.84-1.22] | 1.07 [0.89-1.28] | 1.04 [0.93-1.16] | 1.00 [0.83-1.11] | 0.202 |
| HCY (umol/l) | 10.50 [9.00-12.80] | 10.80 [8.30-13.80] | 10.05 [8.25-11.89] | 12.20 [10.15-16.10] | 0.015 |
| FBG (mmol/l) | 5.36 [4.84-7.02] | 5.17 [4.78-7.31] | 5.20 [4.83-6.69] | 5.30 [4.91-6.96] | 0.644 |
| HbA1C (%) | 6.20 [5.80-7.80] | 6.00 [6.65-7.95] | 6.10 [5.70-7.65] | 6.10 [5.60-6.85] | 0.368 |
| PBG (mmol/l) | 8.15 [6.80-12.34] | 7.28 [6.09-11.06] | 7.65 [6.23-11.63] | 8.10 [6.78-11.41] | 0.747 |

Abbreviations: BMI: body mass index, OSAHS: obstructive sleep apnea-hypopnea syndrome, NIHSS: National Institutes of Health Stroke Scale, mRS: modified Rankin Scale, SBP: systolic blood pressure, DBP: diastolic blood pressure, Hcrt: hypocretin, hsCRP: hypersensitive C reacted protein, ALT: alanine aminotransferase, Cr: creatine, UA: uric acid, TC: total cholesterol, TG: triglyceride; HDL-C: high density lipoprotein cholesterol; LDL-C: low-density lipoprotein cholesterol, HCY: homocysteine, FBG: fasting blood glucose, HbA1C: glycosylated hemoglobin, PBG: postprandial blood glucose.

*p<0.05 was statistical significant.
